# Excess epicardial fat volume in women is a novel risk marker for microvascular dysfunction, which may be a contributing factor in the atypical chest pain syndrome

**DOI:** 10.1186/s43044-021-00159-4

**Published:** 2021-04-13

**Authors:** Mahfouz El Shahawy, Susan Tucker, Lillee Izadi, Antonella Sabatini, Sukanya Mohan

**Affiliations:** 1 Cardiovascular Center of Sarasota, 1950 Arlington Street, Suite 300, Sarasota, FL 34239 USA; 2 Lake Erie College of Osteopathic Medicine, Bradenton, FL USA

**Keywords:** Excess epicardial fat volume, Asymptomatic females, Microvascular disease, Atypical angina

## Abstract

**Background:**

Excess epicardial fat volume (EFV) has been recently implicated in cardiovascular structural and functional abnormalities. It has been associated with abnormal microvascular stiffness (as reflected by radial artery waveform; C2), which may result in microvascular dysfunction and contribute to the atypical chest pain syndrome without obstructive coronary artery disease (CAD). Women have been statistically shown to present with atypical chest pain more often than men and specifically without obstructive CAD. The aim of this study is to assess whether excess EFV in female subjects is associated with significant microvascular dysfunction (i.e., C2), in subjects without obstructive CAD.

**Results:**

We screened 596 asymptomatic subjects, ages 20–79, using the Early Cardiovascular Health Risk Scoring System (ECVHRS), which has been reported. Out of the 596 total subjects, 230 subjects had a CACS. Out of these 230 subjects, 77 subjects (45 females; 32 males) had a 0 CACS. The 45 females from this cohort were the subjects of this study, and they were further categorized into 3 groups: group 1 (normal EFV, non-obese female subjects; *n*=16), females with ECVHRS < 3 and ACC/AHA risk score < 5%; group 2 (*n* = 9), females with elevated EFV and no abdominal visceral obesity; and group 3 (*n*=20), females with elevated EFV and abdominal visceral obesity. The average EFV was determined to be 72±20 cm^3^ among group 1, which indicates the values for normal EFV. The results in group 2 indicate that excess EFV is contributing to the development of microvascular dysfunction, resulting in abnormal micro-arterial (C2) elasticity (*p*< 0.00001), increase in resting blood pressure (*p* =0.0001), an abnormal rise in blood pressure (BP) at rest and post-mild protocol exercise (PME) (*p* = < 0.00001), and abnormal increase in carotid intima-media thickness (CIMT) (*p* = 0.000164).

**Conclusion:**

Excess EFV appears to be not only a novel cardiovascular risk marker, but also the culprit for other cardiovascular risk markers. Based on these findings, elevated EFV may contribute to the development of the atypical chest pain syndrome in females without obstructive CAD. Additionally, EFV is emerging as a potential clinically relevant significant cardiovascular risk biomarker and may become a target to reduce cardiovascular morbidity and mortality.

## Background

Epicardial fat volume (EFV), also referred to as epicardial adipose tissue (EAT), has been recently reported to be a novel cardiovascular risk marker [1–3]. Early detection of excess EFV (i.e., > 69 ± 20 in females and males) has been found to correlate with early cardiovascular structural and functional abnormalities among subjects with various comorbidities, such as type 2 diabetes and obesity, which mandates early treatment through lifestyle modifications and aggressive medical therapy [4]. Epicardial fat belongs to the category of perivascular adipose tissue which also includes the fat surrounding the renal arteries. EFV is unlike abdominal visceral adipose tissue due to its differences in mRNA. Epicardial adipocytes are also smaller than that of abdominal visceral adipocytes [5].

It has been reported that many obese and/or diabetic individuals have increased EFV, which has been associated with many risk factors contributing to coronary artery disease (CAD) [1–3, 6]. Additionally, other studies have found an association between excess EFV and metabolic syndrome [7, 8]. EFV has also been correlated with other independent cardiac biomarkers, such as high levels of C-reactive protein, BNP, and microalbuminuria, along with cardiovascular risk factors (i.e., hypertension, dyslipidemia, and hyperglycemia). It has also been associated with cardiovascular structural and functional abnormalities, such as an abnormal rise in blood pressure post-mild protocol exercise, carotid intima-media thickness, and left ventricular hypertrophy [9].

Under normal physiological conditions, epicardial fat is known to produce anti-inflammatory and anti-atherosclerotic cytokines, such as adiponectin and adrenomedullin, for cardioprotective function including increased free fatty acid oxidation, nitric oxide synthesis, and vasodilation [10–12]. A decrease in nitric oxide availability and vasodilator imbalance has been linked to the development of microvascular disease-based angina [10]. Accordingly, it is time to focus on epicardial fat pathophysiology as an important risk marker for cardiovascular disease (CVD), including atypical chest pain syndrome.

Epicardial fat is defined as the adipose tissue which directly overlies the heart. It can cover 80% of the heart muscle while making up 20% of the heart’s mass [1–3, 5, 13]. Epicardial fat is vascularized by branches of the coronary arteries, and it has no fascia layer separating it from the myocardium; hence, it shares the same microcirculation, suggesting a close and strong interaction with both tissue structures [13]. The physiologic function of epicardial fat is complex and not yet completely understood [5, 13]. There is growing evidence that human epicardial fat produces bioactive cytokines. These cytokines are involved in the regulation of endothelial function, where epicardial fat has been shown to be the strongest predictor of endothelial dysfunction through abnormal local pulse wave velocity in carotid arterial stiffness in menopausal women [14].

Chest pain is the second most common chief complaint in the US emergency departments, accounting for 8 million visits annually. Women have been statistically shown to present with chest pain more often than men [15]. More specifically, once obstructive CAD is ruled out as the cause for this chest pain, microvascular dysfunction makes up 40% of these atypical recurring chest pain diagnoses, particularly in women. This atypical chest pain is referred to as atypical chest pain syndrome and includes a group of syndromes that affect the smaller arterioles in the myocardium, as opposed to the macrovascular coronary arteries. The cause for chest pain in patients with microvascular dysfunction occurs due to reduced blood flow that could be from thickening of the arterioles, from underlying endothelial dysfunction, or from increased resistance in the heart’s microcirculation [15, 16]. The complete pathophysiology of this atypical chest syndrome is still unknown, but it is often attributed to a response from increased myocardial demand due to structural and functional abnormalities.

Excessive accumulation of epicardial fat is also associated with cardiovascular structural and functional abnormalities [1–3, 5] and further increases the workload of the heart through its endocrine/paracrine pathophysiology [17]. Some studies have explored the relationship between epicardial fat and abnormal stress tests or atypical chest pain due to the ability of the epicardial fat to act as a vasoconstrictor in coronary microcirculation [18, 19]. Therefore, it can be further theorized that the pro-inflammatory response hormones induced by excess EFV may result in microvascular dysfunction causing the atypical chest pain often observed in female patients. This study aims to assess whether excess EFV in female subjects is associated with significant microvascular disease, which might ultimately contribute to the atypical chest pain syndrome in subjects without obstructive coronary artery disease.

## Methods

We screened 230 asymptomatic subjects, ages 20–79, for CVD risk using the Early Cardiovascular Health Risk Scoring System (ECVHRS) which consists of 10 tests; 7 of these tests are vascular, and 3 are cardiac. The vascular tests are large (C1) and small (C2) artery stiffness, blood pressure (BP) at rest, and post-mild protocol exercise (PME) consisting of a 3-min walk at 7% elevation and a speed of 2.5 mph. Abnormal BP-PME is defined as a systolic rise of > 30 mmHg compared to systolic BP at rest [20]. Normotension, elevated BP, and hypertension were defined according to the current ACC/AHA guidelines.

Other vascular assessments include carotid intima-media thickness (CIMT), abdominal aorta ultrasound, retinal photography, and microalbuminuria. The 3 cardiac tests are Pro-BNP, ECG, and LVUS [20, 21]. Additional tests included waist circumference, BMI, fasting blood sugar, lipid profile, and hs-CRP. The current ACC/AHA risk score was also calculated to assess and estimate the 10-year risk for the development of ASCVD [22].

**Fig. 1:**
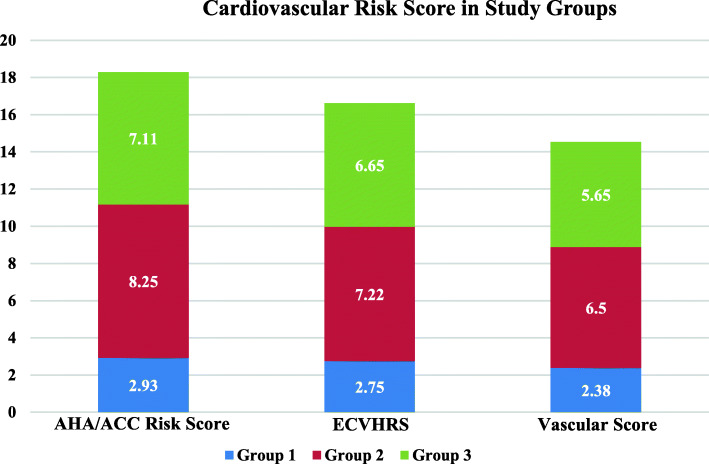
Cardiovascular risk scores in the study groups

These 230 subjects also underwent cardiac CT for CACS and EFV determination using Siemens Somatom Definition Dual source CT scanner 64x2. A total of 77 out of the 230 subjects (45 females; 32 males) had a 0 CACS. These 45 female subjects were then divided into 3 groups: Group 1—16 non-obese female subjects with the following metrics: ECVHRS < 3, ACC/AHA risk score < 5%, EFV between 1 and 95 cm^3^, and CACS 0. Group 1 is used as the control group since the subjects fall under the criterion of low risk according to their ACC/AHA risk score (< 5%), EFV < 95 cm^3^, and having a CACS of 0, which has been shown to implicate a low risk of cardiovascular events or all-cause mortality in the medium and long term [22]. Subjects also had an ECVHRS score of below 3 which is considered low risk [20]. Group 2—9 females who had elevated EFV and no abdominal visceral obesity. Group 3—20 females who had elevated EFV and abdominal visceral adiposity. The respective ACC/AHA risk score, ECVHRS, and Vascular Score for all three groups are seen in Fig. 1. Groups 2 and 3 differ in comparison with each other by BMI and waist circumference, and when compared to low-risk individuals in group 1, it can be implied that regardless of the presence of abdominal visceral obesity, EFV is a significant marker that leads to microvascular disease due to its endocrine functions [23, 24].

### Statistical analysis

The statistical analysis used to determine the statistical significance of the study results included ANOVA, *t* test, and chi-square analysis. A *p* value < 0.05 was considered statistically significant.

## Results

As shown in Table 1, normal EFV in group 1 (72 cm^3^ ± 20 in females) was associated with minimal structural and functional abnormalities. The increased EFV values in group 2 were associated with statistically significant structural and functional abnormalities, particularly C2, as compared with the subjects in group 1. Those in group 3 with increased EFV and abdominal visceral adiposity also depicted significant increases in structural and functional vascular abnormalities that were statistically significant compared to group 1.

**Table 1:** EFV and cardiovascular abnormalities score in study groups

	Group 1 (*n*=16)	Group 2 (*n*=9)	Group 3 (*n*=20)	*p* values between group 2 and group 3	*p* values between group 1 and group 2	*p* values between group 1 and group 3
EFV average (cm^3^)	72 ± 20	113 ± 22	132 ± 28	< 0.0001	.000057	< .00001
Age average (years)	57	64	60	0.12679	Among the 3 groups	Among the 3 groups
BMI average	23.49	27.87	34.92	< 0.0001	Among the 3 groups	Among the 3 groups
Waist circumference (in.)	32.09	35.28	42.15	< 0.0001	Among the 3 groups	Among the 3 groups
AHA/ACC Risk Score (%) average	2.93	8.25	7.11	< 0.02081	.010403	.000314
Early Cardiovascular Health Risk Scoring System (ECVHRS) average	2.75	7.22	6.65	< 0.00015	.000017	.000077
Vascular score average	2.38	6.50	5.65	<0.00015	< .00001	.000036
Abnormal C1 (mL/mmHg × 100)	2 (13%)	4 (44%)	7 (35%)	< 0.00001	< 0.00001	.00027
Abnormal C2 (mL/mmHg × 100)	6 (38%)	5 (55%)	8 (40%)	0.005716	.001725	.303887
Resting BP average (mmHg)	118/69	146/77	135/80	0.000124	.000018	.000459
Abnormal BP rise after PME (mmHg)	3 (20%)	4 (44%)	15 (75%)	< 0.00001	.000275	< 0.00001
Abnormal CIMT	3 (15%)	2 (22%)	8 (40%)	0.000164	.202405	.000075

Group 1: normal subjects; group 2: subjects with elevated EFV, no abdominal visceral obesity; group 3: subjects with elevated EFV, abdominal visceral obesity

*EFV* epicardial fat volume, *BMI* body mass index, *AHA/ACC* American heart association/American college of cardiology, *ECVHRS* Early Cardiovascular Health Risk Scoring System

The results in group 2 indicate the importance of excess epicardial fat in the pathogenesis of microvascular dysfunction, particularly an abnormal micro-arterial (C2) elasticity (*p* < 0.00001) as noted in groups 2 and 3 when compared with the normal group 1. The results in group 2, when compared to the control, indicate the importance of excess EFV even without abdominal visceral adiposity as a major CVD risk factor in women (Table 2).

**Table 2:** Epicardial fat volume and major cardiovascular risk factors

	Group 1 (*n*=16)	Group 2 (*n*=9)	Group 3 (*n*=20)	*p* values
EFV average (cm^3^)	72 ± 20	113 ± 22	132 ± 28	< 0.0001
Resting BP average (mmHg)	118/69	146/77	135/80	0.000124
Total cholesterol average (mg/dL)	207	197	186	0.32476
HDL average (mg/dL)	74	74	52	0.000019
LDL average (mg/dL)	114	103	99	0.411219
Triglycerides average (mg/dL)	92	103	197	0.067938
Fasting blood glucose average (mg/dL)	79	86	105	0.005817
Smoking	0 (0%)	0 (0%)	2 (10%)	

Group 1: normal subjects; group 2: subjects with elevated EFV, no abdominal visceral obesity; group 3: subjects with elevated EFV, abdominal visceral obesity

As shown in Table 3, there was an increase in the amount of microalbumin (a marker of microvascular dysfunction) in the urine of those in groups 2 and 3 when compared to group 1. Although there was no statistical significance between group 3 and group 1 (*p* = 0.06) regarding microalbumin level, there was a greater increase in the value when compared to the control group. Also shown in Table 3 is a statistically significant increase in CRP in groups 2 and 3 when compared to group 1 (*p* = 0.004), further indicating an excess in EFV as a major risk marker for CVD.

**Table 3:** Cardiovascular disease biomarkers in the study groups

	Group 1 (*n*=16)	Group 2 (*n*=9)	Group 3 (*n*=20)	*p* values between groups 2 and 3	*p* values between groups 1 and 3
EFV average (cm^3^)	72 ± 20	113 ± 22	132 ± 28	< 0.0001	< .00001
Microalbumin (mg/mmol)	0.19	0.20	0.38	0.143361	0.056635
BNP average (pg/mL)	83.1	110.11	148.89	0.602817	0.185521
Triglycerides average (mg/dL)	92	103	197	0.067938	0 .027034
C-reactive protein average (mg/dL)	0.19	0.18	0.57	0.004722	0.004127

Group 1: normal subjects; group 2: subjects with elevated EFV, no abdominal visceral obesity; group 3: subjects with elevated EFV, abdominal visceral obesity

## Discussion

**Fig. 2:**
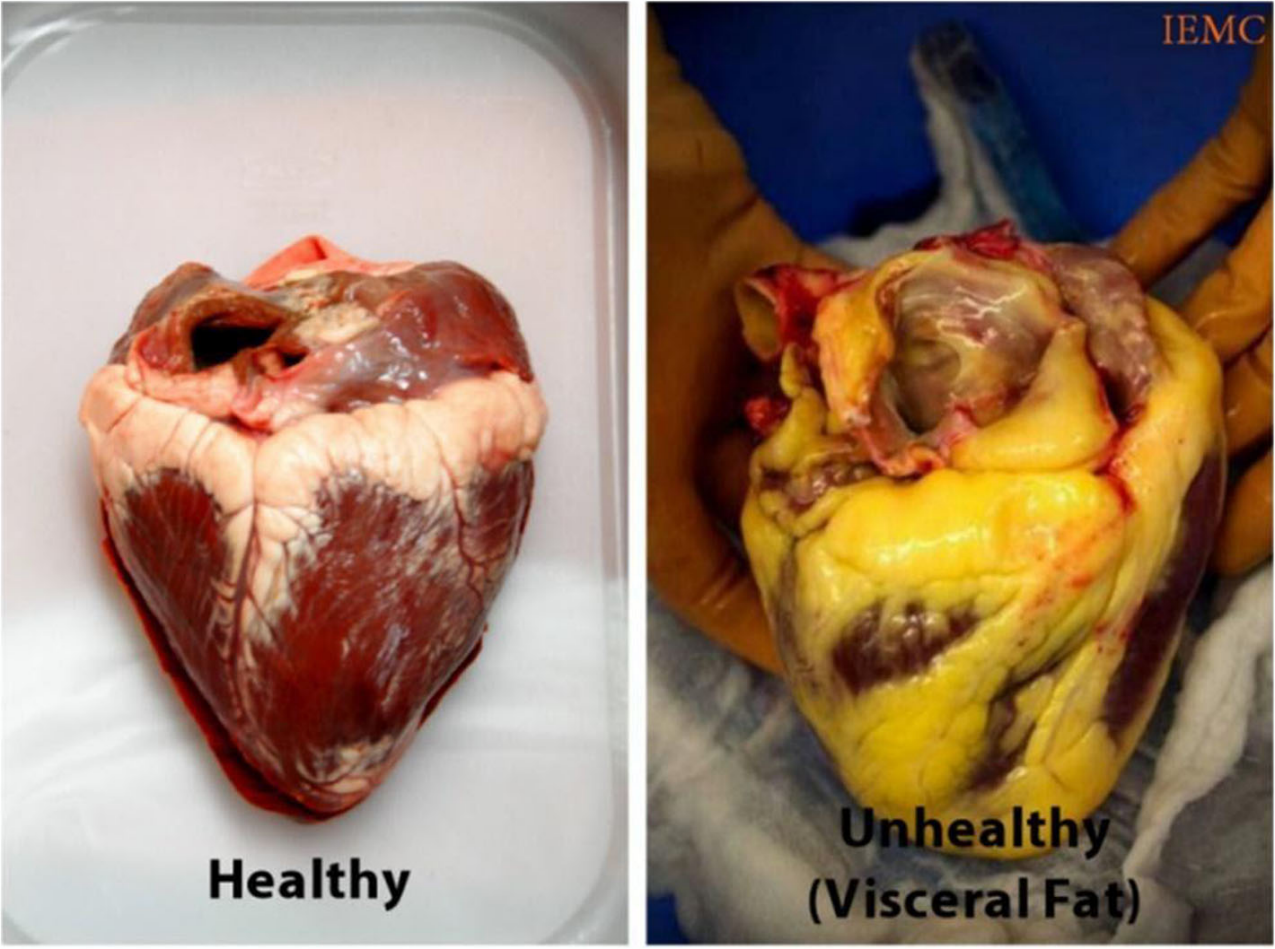
Healthy heart vs. unhealthy heart with excess EFV

Several methods of measuring cardiac fat volumes are feasible, such as total intrathoracic fat volume and epicardial adipose tissue and thoracic fat (i.e., ITFv, EATv, and TF volume) which can be measured directly by non-contrast cardiac computed tomography [3]. Some studies have utilized echocardiography as a way for quantifying EFV [19], but it is more accurately done using a cardiac CT scan. Although previous research has been unable to formulate a definitive gender difference in regard to the impact of epicardial fat volume on cardiovascular risk factors [25], excess EFV might implicate some gender bias in the form of microvascular abnormalities in females rather than a greater prominence of macrovascular abnormalities, leading to atypical chest pain with non-obstructive CAD. Excess EFV, as seen in female subjects with and without abdominal visceral adiposity, is associated with statistically significant increases in cardiovascular risk factors and comorbidities, particularly C2 (Fig. 2). Given the prevalence of obesity among female adults and adolescents in the USA and worldwide [20, 26], we were compelled for a better understanding of the impact that excess EFV has on cardiovascular disease risk, as this can be very important in improving cardiovascular health.

Normal EFV is associated with minimal cardiovascular structural and functional changes, while excess EFV is associated with statistically significant cardiovascular structural and functional abnormalities, as well as the presence of CVD biomarkers, such as C2 abnormalities, abnormal rise in systolic BP, CRP, and microalbuminuria. These microvascular changes have been observed and reported in previous publications over the last decade [20]. The increase in cardiovascular abnormalities can be seen when comparing groups 2 and 3 with normal group 1 subjects, where structural and functional cardiovascular abnormalities are frequently associated with increased EFV. EFV appears to be a significant factor involved in microvascular disease due to its ability to secrete a number of cytokines, referred to as adipokines. These cytokines play an important role in the development of cardiovascular diseases due to their pro-inflammatory properties [23]. Consequently, an increased number of macrophages, T lymphocytes, and mast cells in epicardial fat tissue have been shown in patients with coronary artery disease by several researchers (adipokines source). However, the effect of epicardial fat volume on the manifestation and progression of cardiovascular disease (CVD) and microvascular disease still needs to be more sufficiently and extensively explored.

Upon identification of high-risk individuals with high levels of EFV, an optimal aggressive treatment plan needs to be advised. First and foremost, it is vital to emphasize the importance of weight reduction. Many studies have correlated the regression of epicardial fat with weight loss and weight management, as well as routine exercise (adipokines source). Secondly, consider using statin to reduce LDL levels below 70 mg/dL and utilizing a semaglutide for the reduction of EFV. Weekly administration of either GLP-1 receptor agonists semaglutide or dulaglutide causes a rapid, substantial, and dose-dependent reduction in EAT thickness [4]. Hopefully, these findings will be a stimulus to other investigators to better understand the role of excess epicardial fat and to prevent future cardiovascular complications including microvascular disease.

### Limitations of the study

Only 16 females with 0 CACS and normal-to-low risk ECVDR and ACC/AHA scores were identified and utilized as normal control subjects. Most female subjects were also of Caucasian race, meaning that this study will need to be expanded upon with a cohort of more racial diversity to further the impact of EFV on cardiovascular health. Hopefully, studies soon will have a greater number of subjects and will be able to duplicate and confirm our findings.

## Conclusion

Elevated EFV is associated with significant microvascular abnormalities in females with 0 CACS regardless of abdominal visceral adiposity. These microvascular abnormalities include statistically significant increases in resting blood pressure, abnormal rise in BP-PME, and C2 arterial elasticity. Based on these findings, elevated EFV may contribute to the development of the atypical chest pain syndrome in females without obstructive CAD.

Detection and quantification of excess EFV will help with the early identification and stratification of cardiovascular risk with significant guidance for early application of optimal cardiovascular treatment, including lifestyle modifications and the utilization of newer drugs proven to reduce EFV [6].

As the saying goes: one ounce of early cardiovascular disease prevention is better than pounds of late treatment. *Early detect to protect; the sooner the better.*

## Data Availability

The datasets analyzed during the current study are available from the corresponding author on reasonable request.

## Abbreviation

EFV: Epicardial fat volume
EAT: Epicardial adipose tissue
CAD: Coronary artery disease
CT: Cardiac computed tomography
CVD: Cardiovascular disease
CIMT: Carotid intima-media thickness
C2: A derivative of the radial artery pressure waveform, indicate microvascular dysfunction and predict hypertension and cardiovascular disease
BP-PME: Blood pressure – post-mild exercise protocol
ECVHRS: Early Cardiovascular Health Risk Scoring System
BMI: Body mass index
HDL: High-density lipoprotein
LDL: Low-density lipoprotein
